# Late-onset MELAS syndrome with mtDNA 14453G→A mutation masquerading as an acute encephalitis: a case report

**DOI:** 10.1186/s12883-020-01818-w

**Published:** 2020-06-17

**Authors:** Yuki Yokota, Makoto Hara, Takayoshi Akimoto, Tomotaka Mizoguchi, Yu-ichi Goto, Ichizo Nishino, Satoshi Kamei, Hideto Nakajima

**Affiliations:** 1 Division of Neurology, Department of Medicine, Nihon University School of Medicine, 30-1, Oyaguchi-Kamicyo, Itabashi-ku, Tokyo, 173-8610 Japan; 2 Medical Genome Center, National Center of Neurology and Psychiatry, Tokyo, Japan; 3 Department of Mental Retardation and Birth Defect Research, National Institute of Neuroscience, National Center of Neurology and Psychiatry, Tokyo, Japan; 4 Department of Neuromuscular Research, National Institute of Neuroscience, National Center of Neurology and Psychiatry, Tokyo, Japan; 5 Center for Neuro-infection, Department of Neurology, Ageo Central General Hospital, Saitama, Japan

**Keywords:** MELAS, Late-onset, ND6 gene, Encephalitis

## Abstract

**Background:**

A unique patient with MELAS syndrome, who initially masqueraded as having acute encephalitis and was eventually diagnosed with MELAS syndrome harboring a mtDNA 14453G → A mutation, is described.

**Case presentation:**

A 74-year-old Japanese man was admitted to another hospital due to acute onset of cognitive impairment and psychosis. After 7 days he was transferred to our hospital with seizures and deteriorating psychosis. The results of primary ancillary tests that included EEG, CSF findings, and brain MRI supported the diagnosis of an acute encephalitis. HSV-DNA and antibodies against neuronal surface antigens in the CSF were all negative. With the assistance of the lactate peak on the brain lesions in the magnetic resonance spectroscopy image and genetic analysis of the biopsied muscle, he was eventually diagnosed with MELAS syndrome harboring mtDNA 14453G → A mutation in the ND6 gene.

**Conclusions:**

This case provides a caveat that MELAS syndrome can manifest in the symptoms and ancillary tests masquerading as an acute encephalitis caused by infection or autoimmunity. This is the first adult patient seen to harbor the mtDNA14453G → A with a unique onset, which broadens the phenotypic spectrum of MELAS syndrome associated with ND6 gene mutation.

## Background

Mitochondrial encephalomyopathy with lactic acidosis and stroke-like episodes (MELAS), which is clinically characterized by stroke-like episodes accompanied with headache, seizures, hemiparesis, cortical blindness, hearing disability, and diabetes mellitus [1, 2], is caused by genetic defects in mitochondrial proteins involved in oxidative phosphorylation (OXPHOS) [3]. Previous reports of MELAS have revealed that mutations in both mitochondrial DNA (mtDNA) and nuclear genes essential for mtDNA maintenance are pathogenic [4, 5]. The mtDNA mutations can be categorized into two groups: one type of mutation affects general mitochondrial protein biosynthesis (tRNA and rRNA genes), a representative of which is associated with the nucleotide 3243A → G mutation in the mitochondrial tRNA^Leu^ gene [6]; the other type of mutation causes amino acid substitutions in enzyme complexes involved with OXPHOS (protein-encoding genes) [3, 4].

The dysfunction of respiratory complex I (type I NADH dehydrogenase: ND), which plays an important role in OXPHOS complexes, is considered to be one of the principle mechanisms underlying mitochondrial diseases that include MELAS [7]. An increasing number of pathogenic mutations in ND subunits encoding genes ND1, ND3, ND4, ND5, and ND6 have been described in MELAS [4]. Ravn et al. described the relationship between the 14453G → A mutation within the ND6 gene and severe MELAS in a pediatric patient with myoclonic epilepsy, focal seizure, and ataxia with dystonia [8].

Here we report a unique case of 74-year-old man, whose condition initially masqueraded as acute encephalitis and who was eventually diagnosed with MELAS harboring the 14453G → A mutation.

## Case presentation

A 74-year-old right-handed Japanese man was admitted to another hospital, due to the acute onset of cognitive impairment and psychosis, accompanied by headache and pyrexia. He was treated promptly with intravenous acyclovir. Due to the deterioration of his cognitive function and mental status, he was transferred to our hospital 7 days after the onset of his symptoms. He had normal development and growth before coming of age. His past medical history included hypertension, dyslipidemia, angina pectoris, and chronic renal failure. He had an unremarkable family history, including a lack of neuromuscular diseases, encephalitis, and mitochondrial disease.

**Fig. 1:**
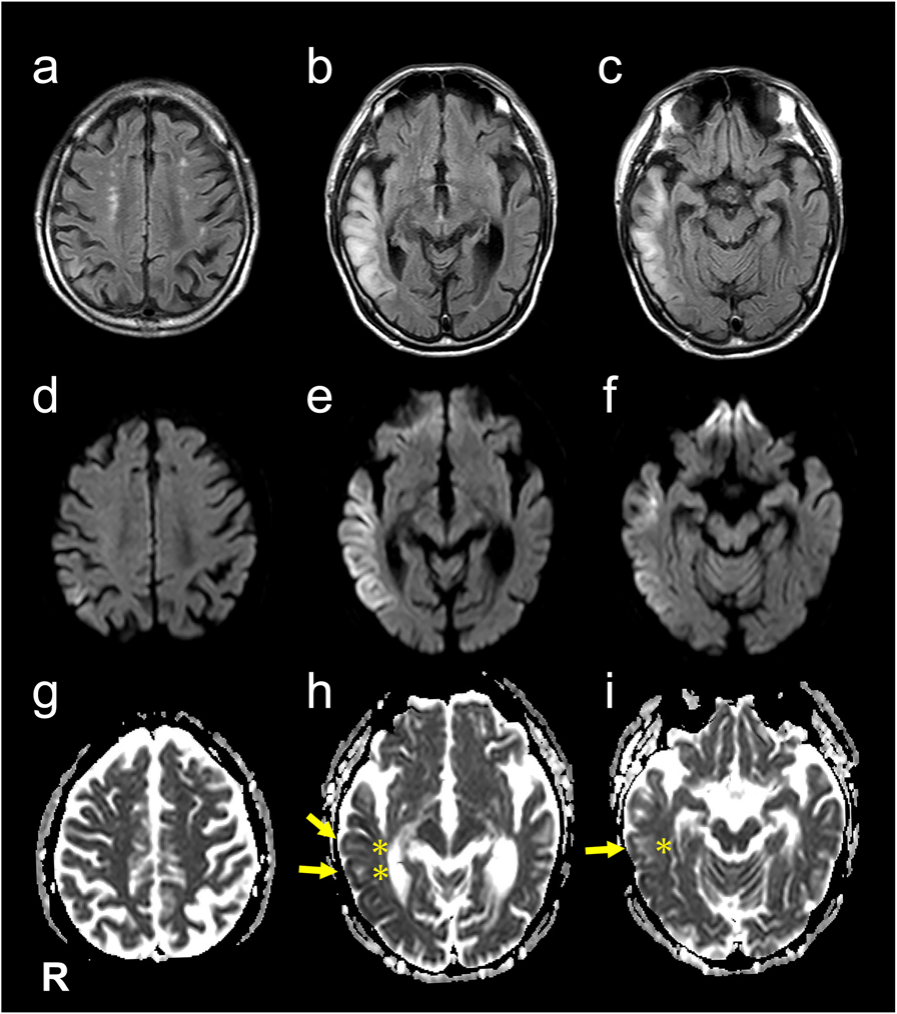
MR imaging findings. Fluid attenuated inversion recovery (FLAIR), (**a**-**c**), diffusion weighted imaging (DWI), (**d**-**f**), and apparent diffusion coefficient (ADC), (**g**-**i**) images of the case at first presentation. FLAIR revealed high-intensity lesions in cortical and subcortical areas of right parieto-temporal lobes. Cortical areas were hyperintense on DWI and hypointense on ADC (arrows). Subcortical areas were hyperintense on DWI and hyperintense on ADC indicating vasogenic edema (asterisks)

**Fig. 2:**
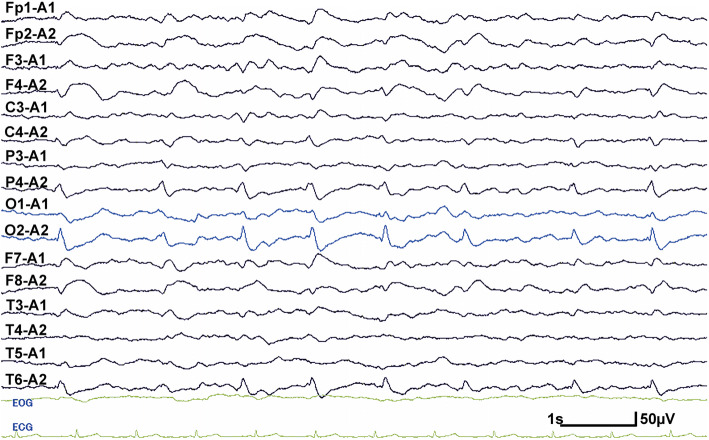
EEG findings. Electroencephalography (EEG) performed on admission revealed poorly organized background activity with periodic lateralized epileptiform discharges (PLEDs), which manifested as high- amplitude, periodic, sharp, transient waves occurring every 1.0–1.5 s(S) over the right temporo-occipital area

At admission, his height was 161.8 cm and weight was 52.6 kg. On physical examination, his body temperature was 38.0 degrees Celsius. A chest auscultation revealed normal respiratory sound and normal heart rate with no murmur. Neurological examination showed mild disturbance of consciousness: GCS 14 (E4 V4 M6), attention disorder, disorientation, psychosis that included abnormal behavior and talkativeness, and left unilateral spatial neglect. On the first day of hospitalization, he presented with no muscle weakness. Ophthalmological examination revealed no abnormal findings in bilateral fundus and oculomotor control. Brain magnetic resonance imaging (MRI) revealed high-intensity lesions in the right parieto-temporal area on fluid attenuated inversion recovery (FLAIR) (Fig. 1 a–c) images and diffusion weighted images (DWI) (Fig. 1 d–f). On the lesions, the apparent diffusion coefficient (ADC) image demonstrated hypo-intensity in the cortical area and high-intensity in the subcortical area (Fig. 1 g–i). A cerebrospinal fluid (CSF) sample included 26 white cells/mm^3^, 70 mg/dL of total protein, and 62 mg/dL of glucose (100 mg/dL of serum glucose). Electroencephalography revealed periodic lateralized epileptiform discharges (PLEDs), which manifested as high-amplitude, periodic, sharp, transient waves occurring every 1.0–1.5 s over the right temporo-occipital region (Fig. 2). We initially diagnosed the patient with acute encephalitis with an infectious (e.g. herpes simplex virus encephalitis; HSVE) or autoimmune origin and continued treatment with acyclovir, while adding intravenous methylprednisolone pulse therapy. On hospital day 4, we discontinued acyclovir due to the receipt of negative real-time PCR results for herpes simplex virus DNA in the CSF. Moreover, autoimmune encephalitis associated with antibodies against neuronal surface antigens (e.g. anti-N-methyl-d-aspartate receptor antibodies) was adequately excluded due to the negative results of autoantibody screening of the CSF with an in-house tissue-based assay using rat brain sections. Moreover, onconeural antibodies including Hu, Yo, Ri, CV2, Ma, amphiphysin, PCA-2, Tr, SOX1, titin, and recoverin all tested negative using line blot assay (EUROIMMUN).

**Fig. 3:**
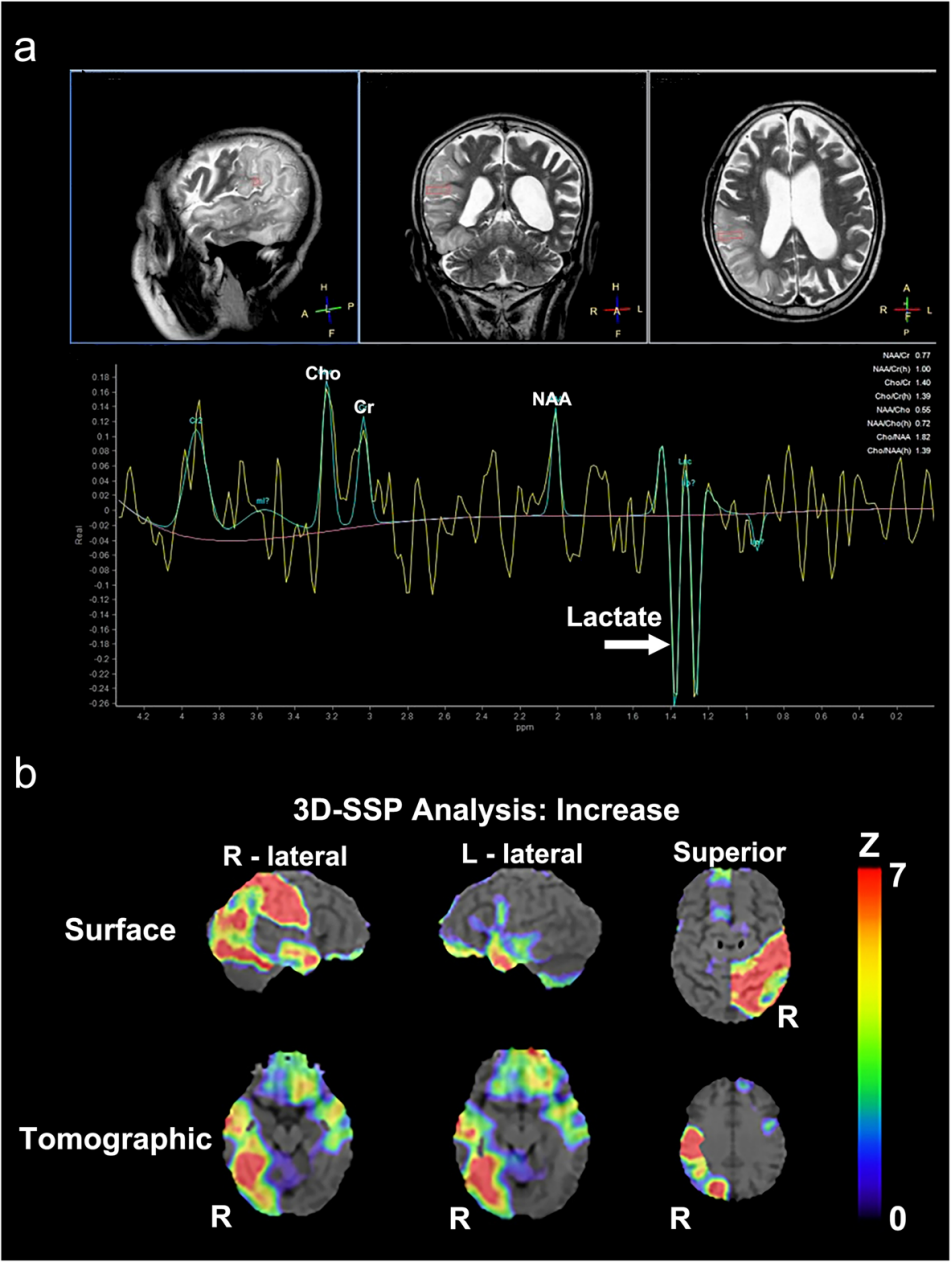
MR spectroscopy and ^123^I-IMP SPECT findings. Magnetic resonance spectroscopy (MRS, A) revealed a markedly elevated lactate concentration, which was detected as an inverted double peak (arrow) in the regions of interest on the high-intensity area within the right parietal lesion; a normal N-acetyl aspartate (NAA) level was also observed. On three-dimensional stereotactic surface projection (3D-SSP) analysis of ^123^I-IMP single photon emission tomography (SPECT, B), regional cerebral blood flow (rCBF) was compared with the blood flow of the normal control group using a Z test. Color-coding represents the statistical significance (Z score) of the increase in rCBF. Z score maps of the brain surface and tomographic views demonstrated that rCBF in the right temporo-occipital area was remarkably increased

**Fig. 4:**
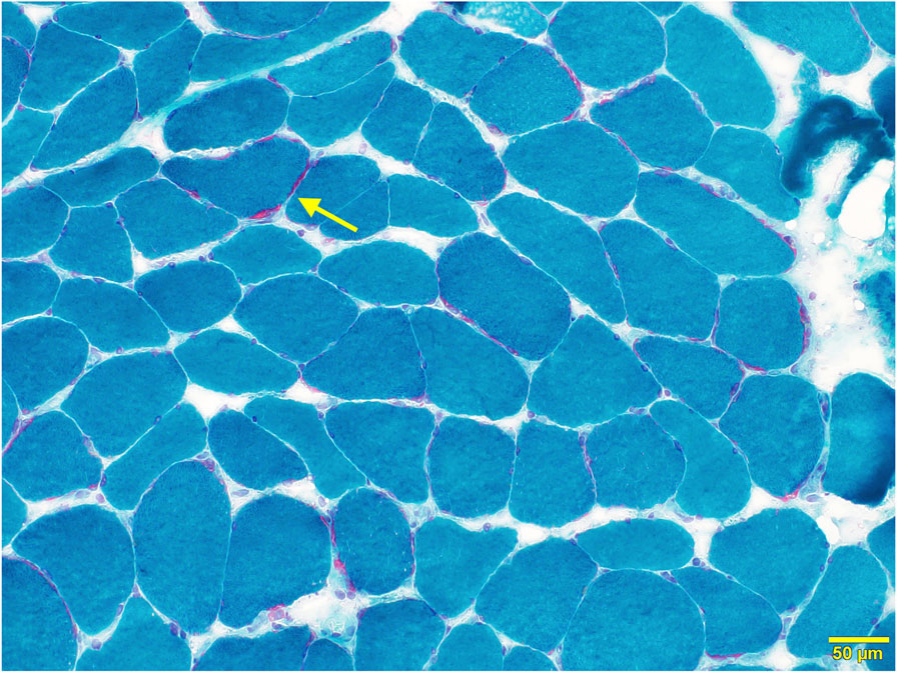
Pathological findings on the biopsied muscle. Modified Gomori-Trichrome staining of the biopsied muscle demonstrated that the specimen had some ragged-red fibers (arrow)

**Fig. 5:**
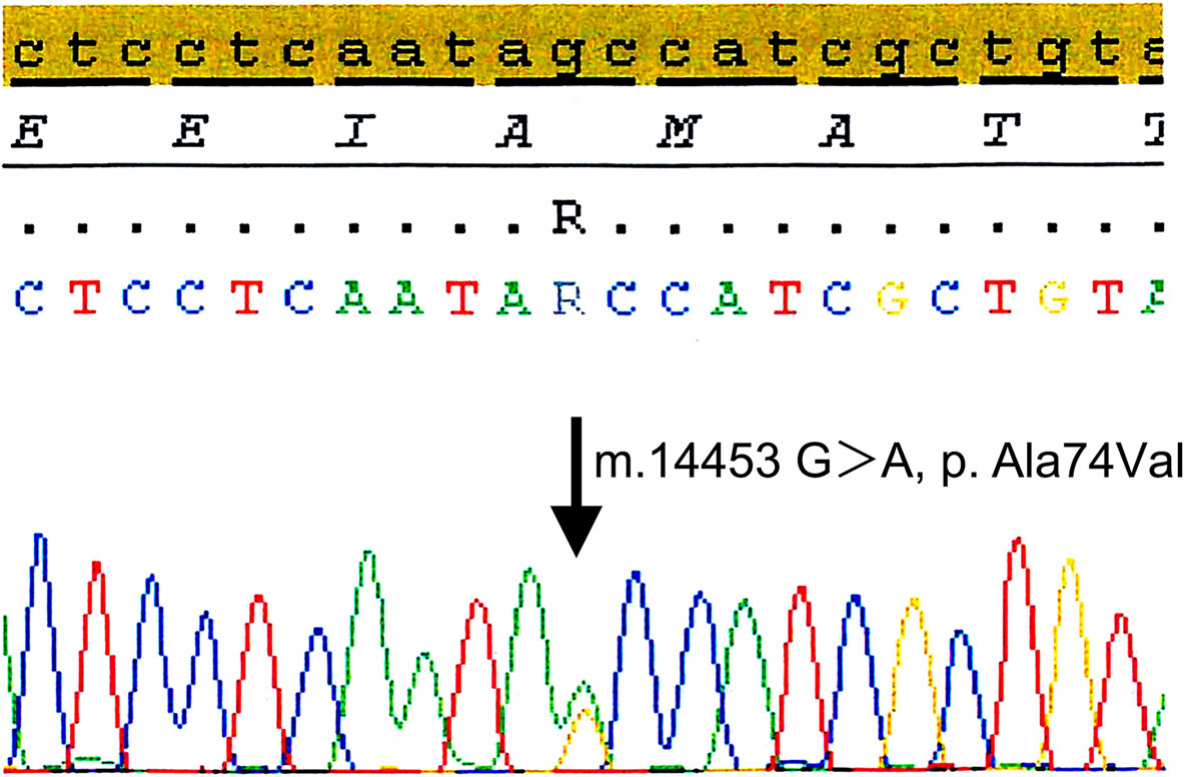
Sequence of part of the patient’s ND6 gene (muscle). Identification of the 14453G > A missense mutation (arrow), which resulted in Ala74Val in the ND6 gene, by sequencing mitochondrial DNA obtained from the biopsied muscle

The patient also developed mild muscular weakness in the left upper and lower extremities after the admission, as evaluated using the Medical Research Council (MRC) Scale, with a score of 3/5. His mental status deteriorated with increasing confusion and aggressive behavior and, therefore, we started a low-dose of continuous intravenous midazolam to sedate the patient. The cortical and subcortical lesions seen on DWI and FLAIR were seen to have progressed to the occipital area on the follow-up MRI. Magnetic resonance spectroscopy (MRS) also revealed an elevated and inverted lactate peak with a normal N-acetyl-aspartate level (Fig. 3a) and ^123^I-IMP single photon emission computed tomography (SPECT) revealed increased uptake in the lesions that were involved on MRI (Fig. 3b). Repeated CSF analyses clarified the elevated level of lactate. These ancillary test results facilitated the possible diagnosis of MELAS, even though the episode was the first neurological event in the patient’s life. With the diagnosis of suspected MELAS, the oral coenzyme Q10 (90 mg/day), L-arginine hydrochloride (7.5 g/day), and vitamin B1 (225 mg/day) were administrated as additional therapies. On day 12, a muscle biopsy of the left biceps brachii was performed for the histopathological diagnosis and analyses of the mitochondrial genetic mutation. Modified Gomori-Trichrome staining of the specimens revealed some ragged-red fibers (Fig. 4) and analyses of the mitochondrial gene revealed an mtDNA 14453G → A point mutation (Fig. 5); this was accompanied by the mtDNA189A → G and 16129G → A point mutations, which were located in the D-loop region of the mtDNA.

With continued treatment, the patient’s disturbed consciousness and mental state gradually improved, but the mild left hemiparesis (MRC scale 4/5 for upper limbs and 4/5 for lower limbs) and cognitive impairment remained (modified Rankin Scale 2). On day 110, he was transferred to the rehabilitation hospital and his Barthel Index score was shown to have improved substantially (from a score of 10 to 80) after intensive rehabilitation. A summary of the clinical course of this patient is shown in Additional Fig. 1.

## Discussion and conclusions

MELAS is, in the main, well characterized to manifest as stroke-like episodes, seizures, and headache. Also, adult patients may frequently have a hearing disability, cortical blindness, or diabetes mellitus [1]. The stroke-like episodes often develop in childhood or adolescence and usually before the age of 40 years [9]. We have described a late-onset stroke-like episode that initially manifested as disturbed consciousness and psychosis with acute febrile seizure. The results of ancillary tests including pleocytosis in the CSF and localized lesions in the lateral temporal lobe on MRI indicated acute encephalitis possible caused by HSVE or an autoimmune event [10]. The negative results for the presence of HSV-DNA and antibodies against neuronal surface antigens in the CSF prompted a further differential diagnosis that included MELAS. The patient was eventually diagnosed with MELAS and was found to harbor a 14453G → A point mutation in mtDNA with the assistance of MRS results and typical pathological findings from biopsied muscle.

Reports of several patients with MELAS who share the syndrome and radiological manifestations of HSVE have been published [11–16], although some extended reviews of the clinical features of molecularly confirmed MELAS did not mention diagnostic confusion with HSVE [2, 17]. The previously reported [11–16] and present cases share clinical characteristics such as relatively late-onset, initial involvement of the temporal lobes and insular lesions on MRI, and immediate induction of ACV. However, all of previous cases harbored a 3243A → G mutation of mtDNA (Table 1). Johns et al., who also described three adult patients with MELAS that initially masqueraded as HSVE, insist that clinical features including the absence of pyrexia, lack of prominent alterations in consciousness, and normal CSF cell count assist in distinguishing MELAS from typical HSVE [11]; in contrast, Gooriah et al. [15] reported a patient with pyrexia, and Hsu et al. [13] described a patient with acute onset pyrexia, headache, and seizures, who showed remarkable pleocytosis in the CSF and unremarkable findings on MRI. The case reported here developed acute cognitive impairment and psychosis, which suggested limbic encephalitis, with pyrexia showing mild pleocytosis at onset. On the other hand, Graus et al. recently reported the clinical diagnostic criteria for autoimmune encephalitis and mentioned that mitochondrial diseases can be offered as a differential diagnosis from autoimmune encephalitis [18]. Indeed, the present case initially met the criteria for ‘possible autoimmune encephalitis’, which is defined in the report, in terms of main symptoms, radiological features, and CSF pleocytosis. Autoantibody screening with an in-house tissue-based assay using the patient’s CSF was valuable enable prompt exclusion of the encephalitis due to the presence of autoantibodies for synaptic proteins. These findings may suggest a caveat for physicians in that MELAS patients can initially share symptoms with those of HSVE or autoimmune encephalitis, showing mild neuroinflammatory signs.

**Table 1:** Literature review of MELAS syndrome masquerading as acute encephalitis

Author (year)	Age/Sex	Symptoms at onset	Focus on MRI	CSF cell (/mm^3^)/ protein (mg/dL)/lactic acid (mg/dL)	Treatment	mtDNA mutation	References
Johns DR (1993)	41/F	Headache, seizure	Lt. temporal lesion with edema and mass effect	ND	ACV, DXA, PHT	3243	11
	44/M	Lt. visual field defect, apraxia of the Lt. limbs, dressing apraxia	Rt. temporoparietal lesion	Acellular/100/ND	ACV, PHT	3243	
	16/F	Confusion, visual difficulties, severe headache, nausea, vomiting	Lt. occipital lesion	2/36/ ND	ACV	3243	
Sharfstein SR (1999)	55/F	Aphasia, delirium	Edema in the Lt. temporal and parietal lobes	3/63/ ND	ACV, PHT	3243	12
Hsu YC (2012)	47/M	Acute onset pyrexia, headache, generalized seizure, agitation	Unremarkable	7750/431.8/ND	CTRX, VCA, VPA → PHT	3243	13
Gieraerts C (2013)	36/M	Anomic aphasia, difficulties of comprehension	Both temporal lobes, insular regions, posterior straight, and cingulate gyri with sparing of the lentiform nuclei	Normal cells/65.8/46.9	ACV	3243	14
Gooriah R (2015)	29/M	Gradual onset of confusion, headache, irritability, personality change, tonic-clonic seizures, pyrexia	Rt. temporoparietal region, Rt. insular cortex and the pole of the Lt. temporal lobe	1/62/ ND	ACV	3243	15
Caldarazzo Ienco E (2016)	45/M	Confusion, headache, generalized seizures	Both temporal lobes	Normal cells/ 83/80	Antiviral, antibiotic therapy, corticosteroid, → carnitine, CoQ10	3243	16
Present case	74/M	Acute cognitive impairment, psychosis, headache, pyrexia	Rt. parieto-temporal areas	26/70/58.0	ACV, mPSL, → CoQ10, L-arginine, Vitamin B1	14453	Present study

*ACV* acyclovir, *CoQ10* coenzyme Q10, *CTRX* ceftriaxone, *DXA* dexamethasone, *F* female, *Lt* left, *M* male, *mPSL* methyl prednisolone, *ND* not described, *PHT* phenytoin, *Rt* right, *VCA* vancomycin, *VPA* valproic acid.

The mutations of the ND6 gene lead to disruption of the mitochondrial respiratory chain involved in the OXPHOS complex, provoking an increase in the sensitivity of complex I to inhibitors binding to the ubiquinone site [19] and drastic reduction in complex I activity [8, 19]. Table 2 lists 16 previously reported pathogenic point mutation sites in the ND6 gene, which are associated with neuromuscular disease [8, 20–33]. According to previous reports, the common clinical manifestation of mutations in the ND6 gene is Leber’s hereditary optic neuropathy (LHON). Several cases presenting with LHON/dystonia, Leigh disease, or MELAS have also been reported. Ravn et al. described a pediatric patient presenting with MELAS, who harbored a mtDNA 14453G → A mutation. A comparison of the clinical features of MELAS with 14453G → A are summarized in Table 3. The mutation load of the mtDNA extracted from biopsied muscle in the report of the previous patient was 82% [8], while that of present case was 53%. In terms of the ‘threshold effect’ theory in mitochondrial disease, Miyabayashi et al. [34] reported that the phenotypic threshold value of mutational load in muscle fibers taken from MELAS patients is 60%. Alternatively, Ng et al. [35] described patients with ND5 point mutation manifesting MELAS or Leigh syndrome at highly variable and relatively low mutational loads of mtDNA extracted from muscle fibers (median 62%, range 28–90%). As the brain is one of the most oxygen-dependent organs reliant mostly on mitochondrial energy supply [36], mitochondrial dysfunctions affect the central nervous system more easily and severely than other tissues. These principles support the suggestion that the mutation load of around 50% from biopsied muscle in the present case could fulfill the phenotypic threshold required to exhibit MELAS, although the heteroplasmy level of the brain tissue, which may be a better predictor of course and severity, are unknown.

**Table 2:** Reported pathogenic mtDNA mutations associated with neuromuscular disease involving the ND6 gene

Nucleotide position and changes	Amino acid changes in ND6	Phenotype	References
14258G → A	P139L	LHON	20
14279G → A	S132L	LHON	21
14325 T → C	N117D	LHON	22
14453G → A	A74V	MELAS	8
14459G → A	A72V	LHON /dystonia	23
14482C → A	M64I	LHON	24
14482C → G	M64I	LHON	25
14484 T → C	M64V	LHON	26
14487 T → C	M63V	Leigh disease	27
14495A → G	L60S	LHON	28
14498 T → C	Y59C	LHON	29
14502 T → C	I58V	LHON	30
14568C → T	G36S	LHON	31
14582A → G	V31A	LHON	20
14596A → T	I26M	LHON /dystonia	32
14600G → A	P25L	Leigh disease	33

*LHON* Leber’s hereditary optic neuropathy, *MELAS* mitochondrial myopathy, encephalopathy, lactic acidosis, and stroke-like episodes, *mtDNA* mitochondrial DNA

**Table 3:** Comparison of the clinical features of MELAS with 14453G → A mutation

Author (year)	Age, y /Sex	Mutation	Locus	Mutation rate Muscle /Blood	Phenotype	Clinical features	mRS Peak /Current	FU (Months)
Ravn (2001)	7/F	5628 T → C 13535A → C 14453G → A	MTTA ND5 ND6	NR NR 82%/78%	MELAS	Myoclonic epilepsy, partial seizure, ataxia with dystonia	5/NR	NR
Present case	74/M	189A → G 14453G → A 16129G → A	D-loop ND6 D-loop	45%/NE 53%/NE 81%/NE	MELAS	Cognitive impairment, psychosis, left hemiparesis	5/2	8

*F* female, *FU* following up period, *MELAS* mitochondrial myopathy, encephalopathy, lactic acidosis, and stroke-like episodes, *M* male, *mRS* modified Rankin Scale, *NE* not examined, *NR* not reported, *y* years

Some studies also described a correlation between the proportion of mutant mtDNA in the affected tissues and the age of onset [37, 38] and also the severity of the disease [37]. On the contrary, Yokota et al. revealed that heteroplasmy at the single-cell level was widely varied among the primary fibroblasts derived from MELAS patients who harbored mtDNA3243A → G [39], which suggests that the mean heteroplasmy level in the affected organ may not represent the disease burden. Further investigation is required to determine why the present case showed later onset of the syndrome and an overall milder clinical course than those of the case reported by Ravn et al. (Table 3). Ravn et al. also argued that the reason their case, which had a mtDNA 14453G → A mutation, caused a severe phenotype is that one or more of the accompanied mutations synergistically modified the pathological phenotype [8]. The present case was found to have a mtDNA 14453G → A mutation in ND6; this was accompanied by the mtDNA189A → G and 16129G → A point mutations, which were located in the D-loop region of the mtDNA. The D-loop region, which is located in the main noncoding area of mtDNA, is involved in replication, transcription, and organization of the mitochondrial genome [40]. Wang et al. described a relationship between pediatric-onset cyclic vomiting syndrome with migraine and a mtDNA 16129G → A mutation [41], although the present case manifested a different syndrome from the one reported. Moreover, the present case also harbored a novel mtDNA189A → G mutation, the pathogenic role of which has not yet been determined [4]. In the present case, mutations in the D-loop regions accompanied by a mtDNA 14453G → A mutation might have modified the syndrome, which consisted of a unique stroke-like episode masquerading as acute encephalitis (MELAS) (Table 3). However, the underlying relationship between the mutation site in the ND6 gene and the clinical phenotypes such as LHON, LHON/dystonia, or MELAS remains to be elucidated.

In conclusion, the present case is a unique example of late-onset MELAS with a 14453G → A mutation accompanied by mutations in the D-loop regions of mitochondrial DNA, the clinical onset of which masqueraded as acute encephalitis with an HSVE or autoimmune cause, which broadens the phenotypic spectrum of MELAS associated with an ND6 gene mutation.

## Supplementary information

**Additional file 1. Summary of the clinical course.** The clinical course of the patient is summarized. The patient developed acute onset psychosis with pyrexia and was transferred to our hospital after deterioration of the syndrome. He also presented with left hemiparesis after admission. Acyclovir (ACV) and intravenous methyl prednisolone were initially administered for acute encephalitis that included herpes simplex virus encephalitis with an autoimmune cause. The findings from magnetic resonance spectroscopy (MRS), which revealed markedly elevated lactate concentration in the regions of interest, assisted the possible diagnosis of MELAS. With a suspected diagnosis of MELAS, oral coenzyme Q10, L-arginine hydrochloride, and Fursultiamine were administered as additional treatments. On day 12, a muscle biopsy of the left biceps brachii was performed for the histopathological diagnosis and analyses of the mutation in the mitochondrial gene, which eventually lead to a confirmed diagnosis of MELAS with a mitochondrial DNA 14453G > A point mutation. The remaining left hemiparesis with gait disability gradually improved. Eight months after onset, the Barthel Index and modified Rankin Scale were substantially improved (from scores of 10 to 80, and 5 to 2, respectively) by intensive rehabilitation with no clinical recurrence.

**Additional file 2.**

## Data Availability

All data and material supporting the conclusions of this article is included in the article. Identifying/confidential information has not been and shall not be shared.

## Abbreviations

ADC: Apparent diffusion coefficient
CSF: Cerebrospinal fluid
DWI: Diffusion-weighted images
EEG: Electroencephalography
FLAIR: Fluid-attenuated inversion recovery
GCS: Glasgow coma scale
HSVE: Helps simplex virus encephalitis
LHON: Leber hereditary optic neuropathy
MELAS: Mitochondrial encephalomyopathy with lactic acidosis and stroke-like episodes
MRC: Medical Research Council
MRI: Magnetic resonance imaging
MRS: Magnetic resonance spectroscopy
mtDNA: Mitochondrial DNA
NADH: Nicotinamide adenine dinucleotide
ND: Nicotinamide adenine dinucleotide dehydrogenase
OXPHOS: Oxidative phosphorylation
PLEDs: Periodic lateralized epileptiform discharges
SPECT: Single photon emission computed tomography
3D-SSP: Three-dimensional stereotactic surface projection
