# Supplementary material for: Late-onset MELAS syndrome with mtDNA 14453G→A mutation masquerading as an acute encephalitis: a case report
Source: BMC Neurol. 2020 Jun 17;20:247. doi: 10.1186/s12883-020-01818-w (PMC7298965; doi:10.1186/s12883-020-01818-w)
Supplement: Supplementary file 2 — Additional file 2. [file 12883_2020_1818_MOESM2_ESM.pdf]

CARE Checklist of information to include when writing a case report

CARE case report guidelines

(cc) BY-NC-ND

Topic	Item	Checklist item description	Reported on Line
**Title**	1	The diagnosis or intervention of primary focus followed by the words "case report"	P1 L1
**Key Words**	2	2 to 5 key words that identify diagnoses or interventions in this case report, including "case report"	P2 L19
**Abstract (no references)**	3a	Introduction: What is unique about this case and what does it add to the scientific literature?	P2 L2-4
	3b	Main symptoms and/or important clinical findings	P2 L5-12
	3c	The main diagnoses, therapeutic interventions, and outcomes	P2 L5-12
	3d	Conclusion—What is the main "take-away" lesson(s) from this case?	P2 L13-16
**Introduction**	4	One or two paragraphs summarizing why this case is unique (**may include references**)	P3 L12 - P4 L3
**Patient Information**	5a	De-identified patient specific information.	P4 L6
	5b	Primary concerns and symptoms of the patient.	P4 L6-7
	5c	Medical, family, and psycho-social history including relevant genetic information	P4 L10-12
	5d	Relevant past interventions with outcomes	P4 L10-11
**Clinical Findings**	6	Describe significant physical examination (PE) and important clinical findings.	P4 L13-18
**Timeline**	7	Historical and current information from this episode of care organized as a timeline	P7 L3-5
**Diagnostic Assessment**	8a	Diagnostic testing (such as PE, laboratory testing, imaging, surveys).	P5 L1-18, P6 L1-18
	8b	Diagnostic challenges (such as access to testing, financial, or cultural)	P5 L1-18, P6 L1-18
	8c	Diagnosis (including other diagnoses considered)	P6 L13-18
	8d	Prognosis (such as staging in oncology) where applicable	P7 L3-5
**Therapeutic Intervention**	9a	Types of therapeutic intervention (such as pharmacologic, surgical, preventive, self-care)	P4 L7, P5 L10, P6 L11-12
	9b	Administration of therapeutic intervention (such as dosage, strength, duration)	P4 L7, P5 L10, P6 L11-12
	9c	Changes in therapeutic intervention (with rationale)	P4 L7, P5 L10, P6 L11-12
**Follow-up and Outcomes**	10a	Clinician and patient-assessed outcomes (if available)	P7 L3-5
	10b	Important follow-up diagnostic and other test results	P7 L3-5
	10c	Intervention adherence and tolerability (How was this assessed?)	Not applicable
	10d	Adverse and unanticipated events	Not applicable
**Discussion**	11a	A scientific discussion of the strengths AND limitations associated with this case report	P7 L8 - P11 L12
	11b	Discussion of the relevant medical literature **with references**.	P7 L8 - P11 L12
	11c	The scientific rationale for any conclusions (including assessment of possible causes)	P8 L2 - P11 L12
	11d	The primary "take-away" lessons of this case report (without references) in a one paragraph conclusion	P11 L13-17
**Patient Perspective**	12	The patient should share their perspective in one to two paragraphs on the treatment(s) they received	Not applicable
**Informed Consent**	13	Did the patient give informed consent? Please provide if requested	**Yes** ■ **No** ☐
